# A dynamic AES cryptosystem based on memristive neural network

**DOI:** 10.1038/s41598-022-13286-y

**Published:** 2022-07-28

**Authors:** Y. A. Liu, L. Chen, X. W. Li, Y. L. Liu, S. G. Hu, Q. Yu, T. P. Chen, Y. Liu

**Affiliations:** 1 State Key Laboratory of Electronic Thin Films and Integrated Devices, University of Electronic Science and Technology of China, Chengdu, 610054 People’s Republic of China; 2 Beijing Microelectronics Technology Institute (BMTI), Beijing, 10076 People’s Republic of China; 3 Nanyang Technological University, Singapore, 639798 Singapore

**Keywords:** Engineering, Physics

## Abstract

This paper proposes an advanced encryption standard (AES) cryptosystem based on memristive neural network. A memristive chaotic neural network is constructed by using the nonlinear characteristics of a memristor. A chaotic sequence, which is sensitive to initial values and has good random characteristics, is used as the initial key of AES grouping to realize "one-time-one-secret" dynamic encryption. In addition, the Rivest-Shamir-Adleman (RSA) algorithm is applied to encrypt the initial values of the parameters of the memristive neural network. The results show that the proposed algorithm has higher security, a larger key space and stronger robustness than conventional AES. The proposed algorithm can effectively resist initial key-fixed and exhaustive attacks. Furthermore, the impact of device variability on the memristive neural network is analyzed, and a circuit architecture is proposed.

## Introduction

The advanced encryption standard (AES), a group symmetric encryption processes with variable key lengths, takes advantage of good security, high efficiency, easy implementation and strong flexibility and has become an international mainstream standard encryption system^1–3^. However, there are still some security problems in AES, such as the fixed initial key, key decoding, and limited key space^4–7^. Chaotic systems were introduced to improve the AES encryption algorithm^8–12^. In 2004, a one-way coupled spatiotemporally chaotic map lattice was used to construct a new AES cryptosystem^8^. In 2018, a novel chaos-based hybrid encryption algorithm design for secure and effective image encryption was presented^9^. In 2019, an image encryption algorithm was proposed based on the combination of a chaos sequence and modified AES^10^. In 2020, a four-dimensional chaotic system was applied to generate keys and improve advanced encryption standard^11^. In 2021, a modified AES cryptosystem with dynamic random keys based on chaos synchronization was presented^12^. There are a few of researches on neural networks for AES^13^; and they mainly focus on optimization and searching problems. Hopfield et al. introduced the energy function to a neural network to solve the travelling salesman problem (TSP)^14^. Multilayer perceptron neural networks (MLP NNs) were trained for sonar dataset classification^15,16^.

In addition, the abovementioned algorithms improve AES without considering physical implementations. As the fourth fundamental circuit component, memristors have many advantages^17^, such as nonlinearity, memory properties, low power consumption, and simple structures^18^. There is also much research on building chaotic systems and neural networks based on memristors^19–31^. In 2008, several nonlinear oscillators were derived from Chua's oscillators by replacing Chua's diodes with memristors^19^. In 2012, a delayed switching effect was used to control the switching of a memristor synapse between two neurons^21^. In 2020, a physical memristor based on the Muthuswamy-Chua chaotic system (circuit) was provided^29^. These memristive neural networks are rarely applied to encryption systems based on lightweight cryptography. Jack Cai presented a cryptography architecture based on memristor crossbar array, binary hypervectors, and neural network^32^. A hardware module based on memristor devices was demonstrated for AES key generation^33^. Some applications in image encryption were found by the memristor based chaotic system^34^.

In this paper, based on the memristor-based transient chaotic neural network (MTCNN)^35^, a long-time chaotic state is realized by altering the value of the parameters. By MTCNN, the AES initial key is dynamically generated to realize "one-time-one-secret" encryption. Simultaneously, to improve security, Rivest-Shamir-Adleman (RSA) encryption is used to encrypt the initial parameters of a chaotic network. The histogram analysis of image encryption, sensitivity and statistics analysis have been carried out, and the capability and improvement in this proposed AES cryptosystem have been examined. At the same time, we also tested the variability of the device and the noise immunity of the network, and the results proved that MTCNN has good anti-interference characteristics. Finally, the circuit level architecture is proposed and simulated successfully, which proves it can be implemented in hardware.

## Background and methodology

### AES

Rijndael was chosen as the advanced encryption standard by the National Institute for Standards and Technology (NIST) because of its elegance, efficiency and security in 2000. AES is a symmetric encryption algorithm with a block length of 128-bit. The numbers of encryption rounds are related to the key lengths and are 10 rounds for a 128-bit key, 12 rounds for a 192-bit key and 14 rounds for a 256-bit key. Each round of AES encryption mainly includes SubBytes, ShiftRows, MixColumns and AddRoundKey. AddRoundKey, which applies XOR operation between the input state matrix and the key, is the most important step. The traditional AES system generates each round key by a fixed key expansion.

### MTCNN

MTCNN is designed and implemented by introducing a memristor into a transient chaotic neural network (TCNN), which has great self-control when switching between chaotic and steady states, and it is described as^35^:

1 $$\begin{array}{c}{\mathrm{x}}_{i}\left(t\right)=\frac{1}{1+{e}^{{-y}_{i}(t)/\varepsilon }}\end{array}$$

2 $$\begin{array}{c}{y}_{i}\left(t+1\right)=k{y}_{i}\left(t\right)+\alpha \left(\sum_{j=1}^{n}{w}_{ij}{x}_{j}\left(t\right)+{I}_{i}\right)-{z}_{i}\left(t\right)\left({x}_{i}\left(t\right)-{I}_{0}\right)\end{array}$$

3 $$\begin{array}{c}{z}_{i}\left(t\right)=b\cdot \frac{1}{\sqrt{{{M}_{0}}^{2}+{2k}_{m}\varphi }}\end{array}$$

4 $$\begin{array}{c}\frac{d\varphi }{dt}=c\cdot {\mathrm{x}}_{i}\left(t\right)\end{array}$$

where ${\mathrm{x}}_{i}$ is the output of neuron $\mathrm{i}$, ${y}_{i}$ is an internal state of neuron $\mathrm{i}$, ${z}_{i}$ is the self-feedback connection weight of neuron $\mathrm{i}$ (${z}_{i}$>0), ${w}_{ij}$ is the connection weight between neuron $\mathrm{j}$ and neuron $\mathrm{I}$, $\alpha$ is a positive scaling parameter for inputs ($\alpha$>0), $k$ is a damping factor of the neuronal membrane (0 < $k$<1), $\varepsilon$ is a steepness parameter of the output function ($\varepsilon$>0), ${M}_{0}$ denotes the initial resistances of the memristor, $b$ and $\mathrm{c}$ are the scaling parameters, $\mathrm{\varphi }$ is the magnetic flux of the memristor, $\frac{d\varphi }{dt}=c\cdot {\mathrm{x}}_{i}\left(t\right)$, and ${k}_{m}$ is the initial value of the memristor.

**Figure 1:**
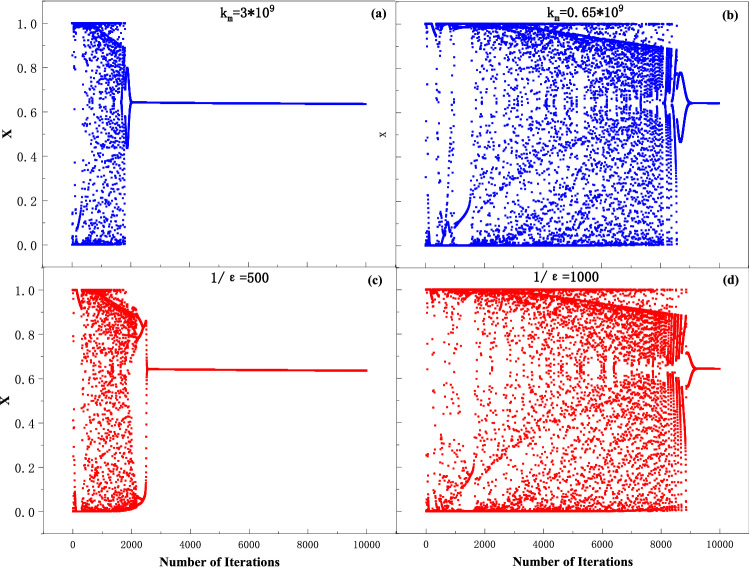
The period of the chaotic state of MTCNN controlled by parameter value. (**a**) ${k}_{m}$ = 3*10^9^; (**b**) ${k}_{m}$ =0.65*10^9^; (**c**) $1/\upvarepsilon$ = 500 and (**d**) $1/\upvarepsilon$ = 1000.

The settings of various parameters can be found in^35^. ${\mathrm{M}}_{0}=1100,\mathrm{k}=0.9, \frac{1}{\upvarepsilon }=800,{I}_{0}=0.65,{k}_{m}={10}^{9}, b=100, c=1.25\times {10}^{-5},\mathrm{ and }{y}_{0}=0.5$. This work aims to improve the AES algorithm with MTCNN. It requires prolonging the chaotic state of the system to generate chaotic sequences. As shown in Fig. 1, the period of the chaotic state can be controlled by the parameters. When increasing $\upvarepsilon$ or decreasing ${k}_{m}$, the duration of chaos becomes significantly longer. Figure 1 shows that the number of iterations of chaos could increase from 2000 to 8500 within $1/\upvarepsilon$ from 500 to 1000 or ${k}_{m}$ from 3 × 10^9^ to 0.65 × 10^9^.

### Dynamic AES using MTCNN

**Figure 2:**
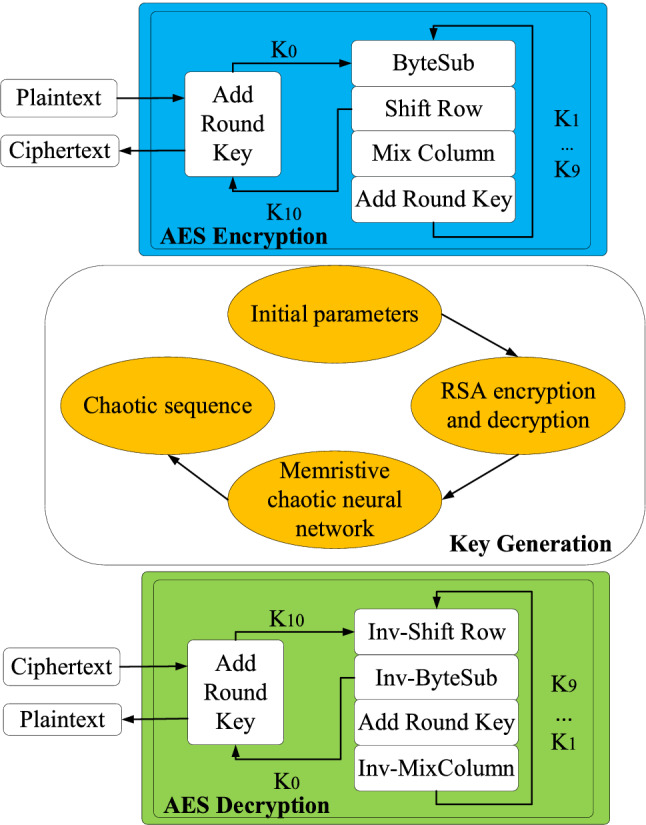
Schematic illustration of the proposed AES encryption and decryption process based on MTCNN.

The MTCNN model is introduced to change the encryption key every round, as shown in Fig. 2. The plaintext is encrypted with AES, and the parameters of the key generated by the chaotic neural network are encrypted with RSA. The chaotic sequence generated by MTCNN can be used as a key for each round of encryption and decryption.

**Figure 3:**
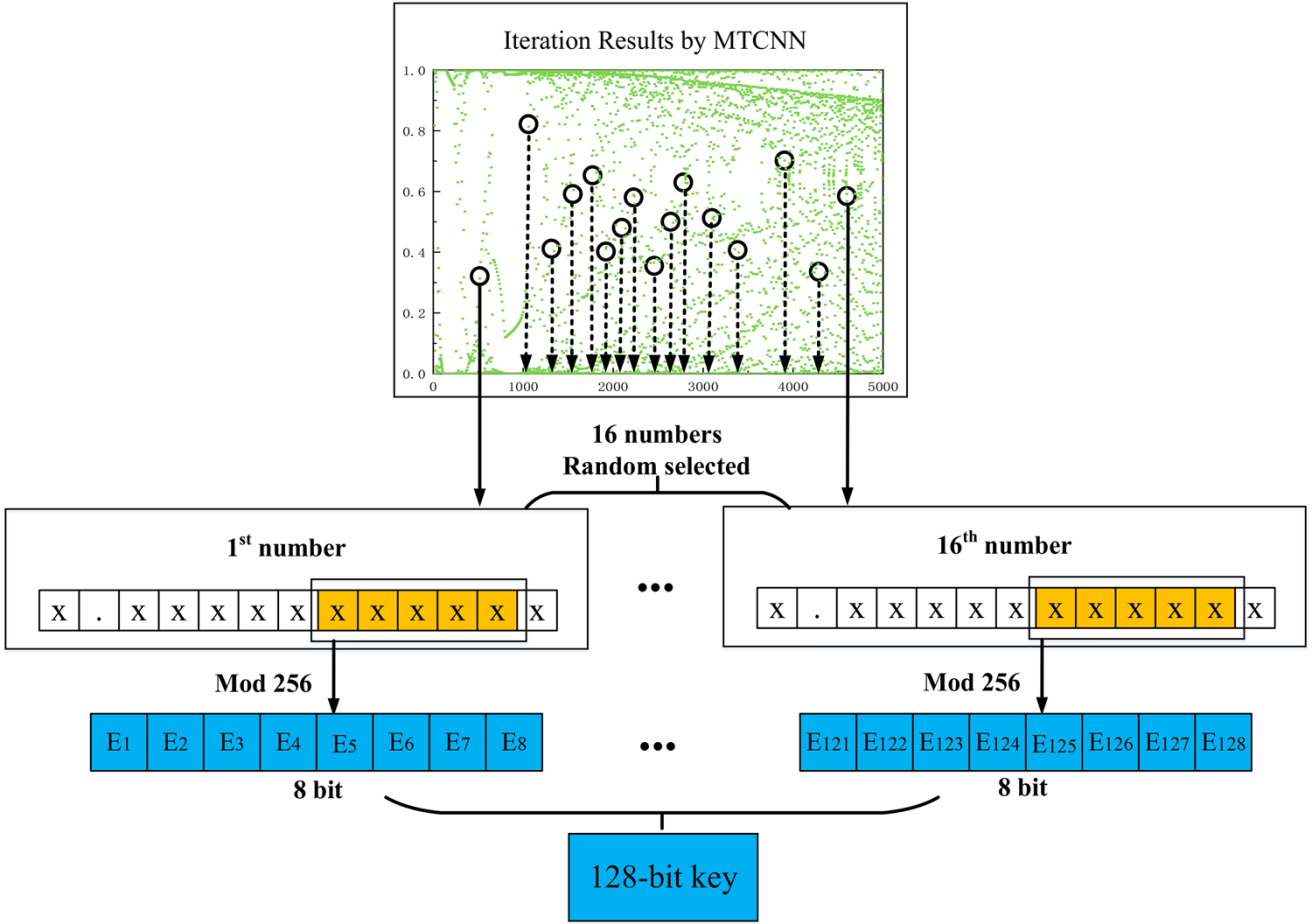
Schematic diagram of the 128-bit key generation by MTCNN.

The chaotic sequence generated by MTCNN realizes key generation through the following process, as shown in Fig. 3. Firstly, 16 floating-point numbers with values between 0 and 1 are randomly selected by 16 iterations in the chaotic period. Then, 6 to 10 digits after the decimal point of every floating-point number are taken, and are divided by 256 to obtain an integer in [0, 255]. By converting the decimal system into a binary system, each integer in [0, 255] is converted into an 8-bit binary number, and finally, 16 integers can be used to obtain a 128-bit sequence, i.e., a 128-bit key.

As a chaotic neural network is sensitive to the initial value, with a slight modification in the initial value each time, a different nonduplicate key sequence can be obtained and conformed to the key standard. The key generated from each initial value can be used for one round of AES encryption, and ten initial values can complete one round AES block encryption.

## Results and analyses

We examine image and text encryption and decryption by the proposed algorithm. The encryption parameters are set as mentioned above.

### Histogram analysis of image encryption

**Figure 4:**
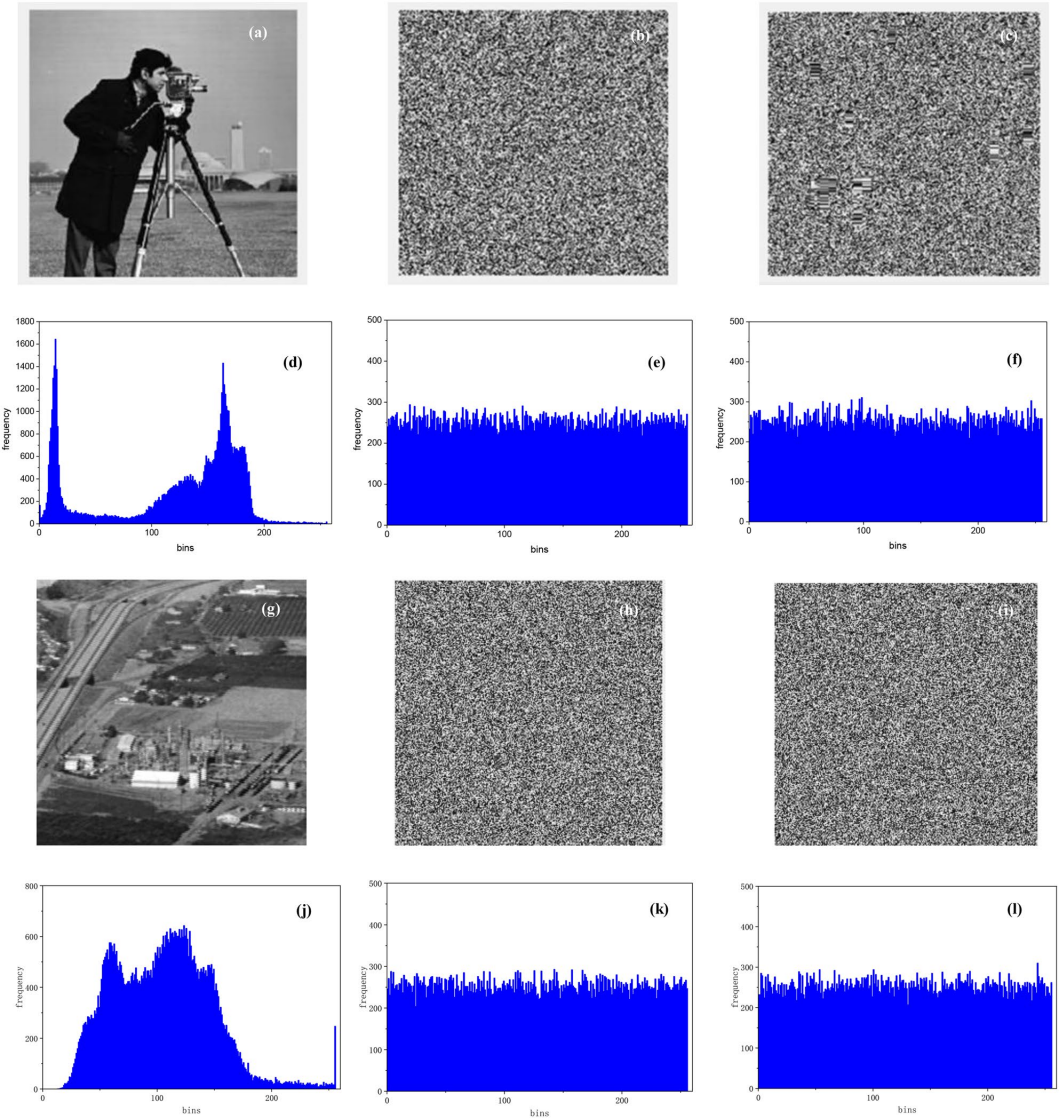
Histograms of the image encryption. (**a**-**f**) Cameraman: (**a**) original image; the image after (**b**) proposed encryption, (**c**) conventional AES; histogram of the (**d**) original image, (**e**) encrypted by proposed algorithm, (**f**) encrypted by conventional AES; (**g**-**l**) Chemical plant: (**g**) original image; the image after (**h**) proposed encryption, (**i**) conventional AES; histogram of the (**j**) original image, (**k**) encrypted by proposed algorithm, (**l**) encrypted by conventional AES.

As shown in Fig. 4, this system successfully realizes the encryption and decryption of the greyscale images (256 $\times$ 256) "Cameraman" and "Chemical plant". Their histograms before encryption and after encryption by the proposed AES model and conventional AES model are presented. The histograms of the original images have a nonuniform distribution and vary widely, while the pixel values of the encrypted images are distributed uniformly. Compared with conventional AES, the proposed algorithm has better balance and smaller variations. This demonstrates that the proposed algorithm has better security and can resist statistical attacks more efficiently. However, the computing time of the proposed algorithm is a little long due to the high complexity.

### Sensitivity analysis

The experiment on the sensitivity of the proposed cryptosystem is performed by altering the initial values of the memristive neural network parameters. After changing the parameters (${\mathrm{M}}_{0},\upvarepsilon ,{I}_{0},{k}_{m},\, and \, {y}_{0}$) slightly, the bit change rate of ciphertext is shown in Table 1. In the table, when ${\mathrm{M}}_{0},\upvarepsilon ,{I}_{0},{k}_{m}$ and ${y}_{0}$ are varied about $1\times {10}^{-16}$, each of them can obtain a bit change rate of ciphertext of approximately 50%, which means the key avalanche phenomenon is apparent. This verifies the good sensitivity, key dependence and effectiveness of the algorithm.

**Table 1:** Bit change rate of ciphertext with the change of parameters.

Parameter	Initial value	New value	Bit change rate of ciphertext (%)
${M}_{0}$	1100	1100 + 1100 × 10^–16^	47.66
${k}_{m}$	10^9^	10^9^+ 10^9^ × 10^–16^	50.98
$1/\varepsilon$	800	800 + 800 × 10^–16^	46.68
${I}_{0}$	0.65	0.65 + 0.65 × 10^–16^	50.10
${y}_{0}$	0.5	0.5 + 0.5 × 10^–16^	51.46

### Statistics analysis

**Figure 5:**
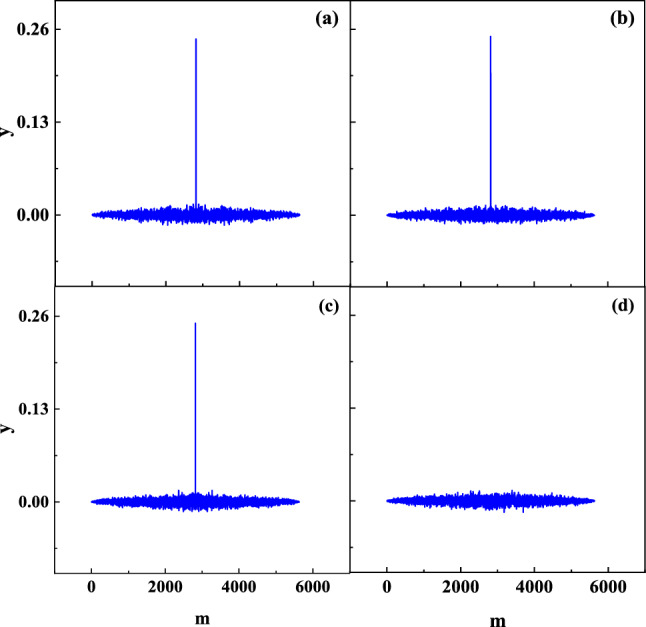
Correlation detection of encryption algorithm based on MTCNN. The autocorrelation function for (**a**) the chaotic sequence, (**b**) the ciphertext, (**c**) the plaintext; and (**d**) the cross-correlation function values of plaintext and ciphertext.

The correlation detection of the proposed encryption algorithm is shown in Fig. 5. To calculate the autocorrelation and cross-correlation, the following equations are used^36^:

5 $${R}_{{S}_{1},{S}_{2}}\left(m\right)=\frac{1}{N}\sum_{i=0}^{N-1}[{S}_{1}\left(i\right)-\overline{{S}_{1}}][{S}_{2}\left(i+m\right)-\overline{{S}_{2}}]$$

where ${R}_{{S}_{1},{S}_{2}}$ is the cross-correlation of sequences ${S}_{1}$ and ${S}_{2}$; $\overline{{S}_{1}}$ and $\overline{{S}_{2}}$ are the sequence means; and $m$ is the correlation interval. When ${S}_{1}={S}_{2}$, the above equation becomes an autocorrelation function. It can be confirmed that the chaotic sequence generated by MTCNN has good randomness, as shown in Fig. 5a. When $m=0$, the autocorrelation function is equal to 0.25, and when $m$ is not equal to 0, the autocorrelation function tends to be zero. The whole autocorrelation function is close to the δ function and is quasi-random. Moreover, by measuring the frequency of the chaotic sequence, the average value of the balance degree of the chaotic sequence is 49.76%. This means that the "0" and "1" sequences of the chaotic sequence are evenly distributed. The balance degree of the chaotic sequence is good, as shown in Fig. 5a–d. It can also be seen that the values of the autocorrelation sidelobe and cross-correlation function of the ciphertext sequence are close to zero, indicating that the ciphertext encrypted by MTCNN shows good randomness and is not related to plaintext.

Theoretically, the key space of conventional "AES128" is ${2}^{128}\approx 3.4\times {10}^{38}$. The memristive neural network used in this work can act as the parameter of the key, and the parameter type and range of the proposed MTCNN method are presented in Table 2. The total key space size is equal to the product of all parameter spaces: $\mathrm{K}={K}_{{M}_{0}}\times {K}_{k}\times {K}_{\varepsilon }\times {K}_{{I}_{0}}\times {K}_{{y}_{0}}\times {K}_{{k}_{m}}\times {K}_{b}\times {K}_{c}\approx 8.62\times {10}^{123}$, where ${K}_{{M}_{0}},{ K}_{k},{ K}_{\varepsilon }, {K}_{{I}_{0}},{ K}_{{y}_{0}},{ K}_{{k}_{m}},{ K}_{b}\mathrm{ and }{K}_{c}$ are the key spaces for the parameters in Table 2. The key space of the proposed AES cryptosystem is much larger than that of the conventional AES system.

**Table 2:** List of parameter types and range of the proposed MTCNN.

Parameter	Data type	Value range	Space size
${M}_{0}$	Double	[400,2000]	${K}_{{M}_{0}}\approx 1600\times {10}^{16}$
$k$	Double	[0.45,0.99]	${K}_{k}\approx 0.54\times {10}^{16}$
$1/\varepsilon$	Int	[400,1000]	${K}_{\varepsilon }\approx 600$
${I}_{0}$	Double	[0.6,0.7]	${K}_{{I}_{0}}\approx 0.1\times {10}^{16}$
${y}_{0}$	Double	[0.1,0.9]	${K}_{{y}_{0}}\approx 0.8\times {10}^{16}$
${k}_{m}$	Double	[0.$1\times {10}^{9}$,$10\times {10}^{9}$]	${K}_{{k}_{m}}\approx 9.9\times {10}^{9}\times {10}^{16}$
$b$	Double	[50,150]	${K}_{b}\approx 100\times {10}^{16}$
$c$	Double	[$9\times {10}^{-6}$,$3\times {10}^{-5}$]	${K}_{c}\approx 21\times {10}^{-6}\times {10}^{16}$

**Figure 6:**
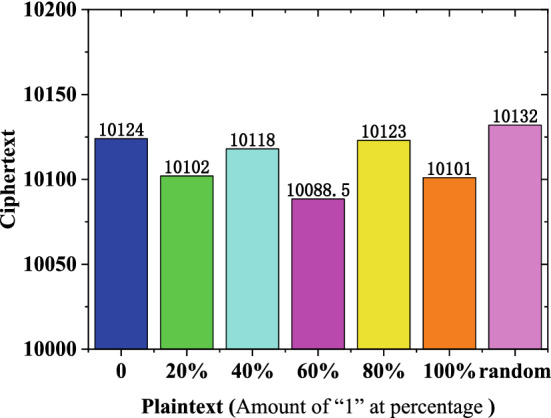
Frequency detection of the encryption algorithm based on the proposed MTCNN.

Figure 6 shows the frequency detection for encryption with MTCNN. It should be noted that regardless of whether the plaintext has prominent statistical characteristics or randomness, the corresponding ciphertext has good randomness. This means that the ciphertext does not depend on the statistical characteristics of the plaintext. The algorithm has good plaintext independence and can effectively resist differential attacks. Furthermore, 20 poker tests were carried out, and the test results are shown in Table 3. According to^36^, the 20,000-bit random ciphertext encrypted by AES based on MTCNN is divided into 4-bit groups, and $f(i)$, the number represented by the 4-bit group elements, is counted. The statistic X is calculated by Eq. (6), which passes when 2.16 < X < 46.17.

**Table 3:** The results of 20 times of Poker test.

Test Results of statistic X			
13.34	19.18	23.93	8.46
14.61	17.29	18.64	14.57
20.33	10.62	9.66	27.28
15.87	16.72	12.06	18.98
21.36	13.54	17.22	17.09

6 $$\begin{array}{c}X=\frac{16}{5000}\times (\sum_{i=0}^{15}[{f\left(i\right)}^{2}])-5000\end{array}$$

The results show that X falls in the range of [8.46, 27.28], and its average value is 16.54 ∈ [2.16, 46.17]. The ciphertext of this algorithm has good randomness and can resist attacks, such as statistical analysis attacks.

### Hardware simulations

**Figure 7:**
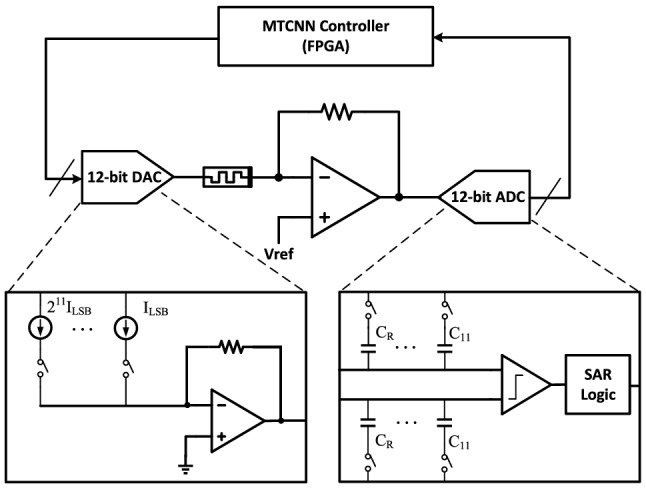
Hardware implementation schematic of the MTCNN system.

As shown in Fig. 7, the proposed circuit level architecture has four modules, including MTCNN controller, 12-bit digital-to-analog converter (DAC), 12-bit analog-to-digital converter (ADC), and memristive amplifier. MTCNN controller is mainly used to complete the iterative algorithm of chaotic system, and to constantly output voltage excitation. DAC and ADC, using the 0.13-um COMS technology, are applied for the conversion between MTCNN controller and device-level circuit. (Pt/TiO_2_/Pt) memristors architecture are fabricated on top of CMOS in our laboratory. The CMOS and memristors are integrated by the hybrid technology. The working process is described as follows. Firstly, the initial output of MTCNN controller is converted into a voltage through 12-bit DAC. Then the current obtained by the memristor, through the amplifier circuit is convert to a voltage. Finally, the output voltage is sent back to MTCNN controller through ADC for calculation, and the next voltage is output.

This circuit involves many conversions between analog and digital. The precision of the convertors has an important effect on working of the design. In addition, a small error from quantization accuracy may be continuously accumulated by the iterative process in the circuit, possibly leading to final wrong result. Therefor a 12-bit resolution DAC and ADC are very necessary for this work. In this work, both DAC and ADC are designed with conventional architecture. The current-steering DAC is designed based on an array of matched current sources which are binary decoded. Each switch of different weights is controlled by the input digital code and decides the magnitude of the current in each branch. Finally the output of the DAC is obtained by the summing circuit. The successive approximation register (SAR) ADC consists of sample-and-hold (S/H), comparator, DAC, and SAR logic control circuit. The switch procedure is realized with a binary search algorithm. That means that the input signal is compared with the reference voltage output by the DAC from the most significant bit (MSB) to the least significant bit (LSB). When input the signal, the switch of largest capacitor C_R_ is turn on and the other capacitors are turn off. The first comparison is done by the comparator. If the input voltage is higher than the reference voltage, MSB is "1". Otherwise, it is "0". The switch of the largest capacitor becomes turn-off. Then we repeat the switch procedure until the LSB is approached. That means the final output digital code value is obtained. Each comparison and conversion are controlled by the clock signal generated by the SAR logic control circuit.

**Figure 8:**
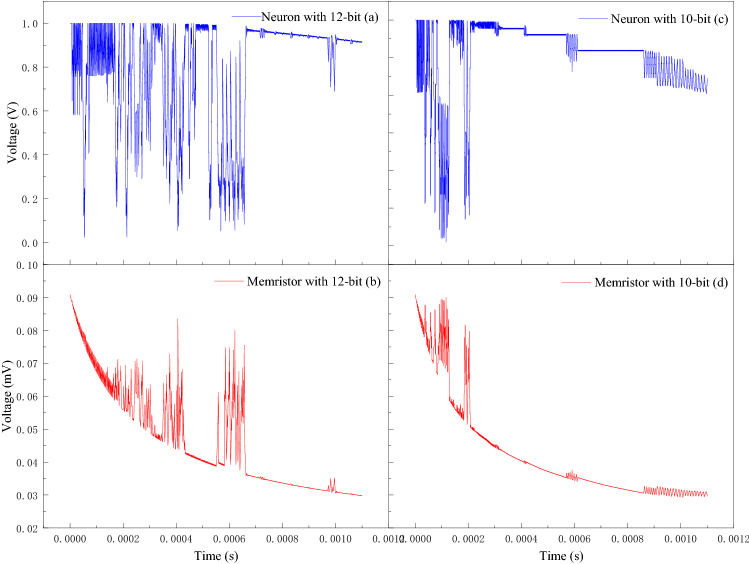
Hardware simulation results. (**a**) and (**b**) are the output voltage of the neuron and memristor with 12-bit ADC/DAC, respectively; (**c**) and (**d**) are the output voltage of the neuron and memristor with 10-bit ADC/DAC.

The results of circuit simulation based on 12-bit and 10-bit DAC/ADC are illustrated in Fig. 8. Both 12-bit and 10-bit DAC/ADC enables the chaos, which proving that the proposed MTCNN can be implemented in hardware. Furthermore, it obviously has richer chaotic dynamic characteristics with the 12-bit ADC/DAC than 10-bit, and its chaotic time is longer, which will increase the complexity and improve the security of encryption cryptosystem.

## Discussion

**Figure 9:**
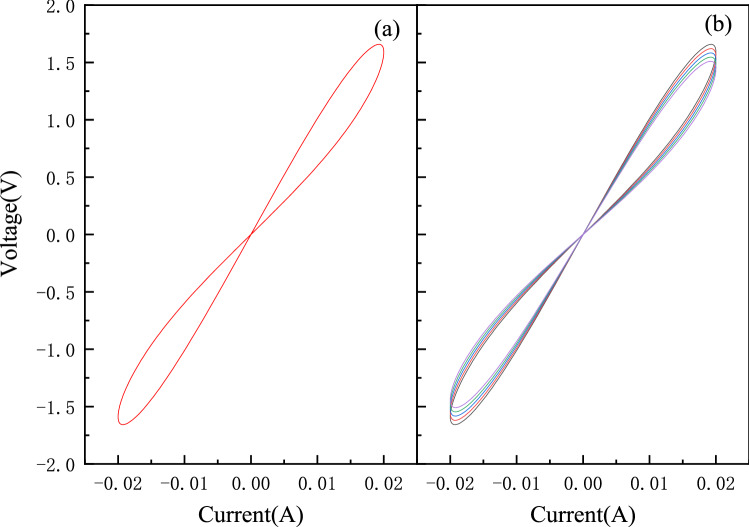
current–voltage (I-V) characteristics of a memristor. (**a**) an ideal HP memristor; (**b**) conductance drift of the memristor.

**Figure 10:**
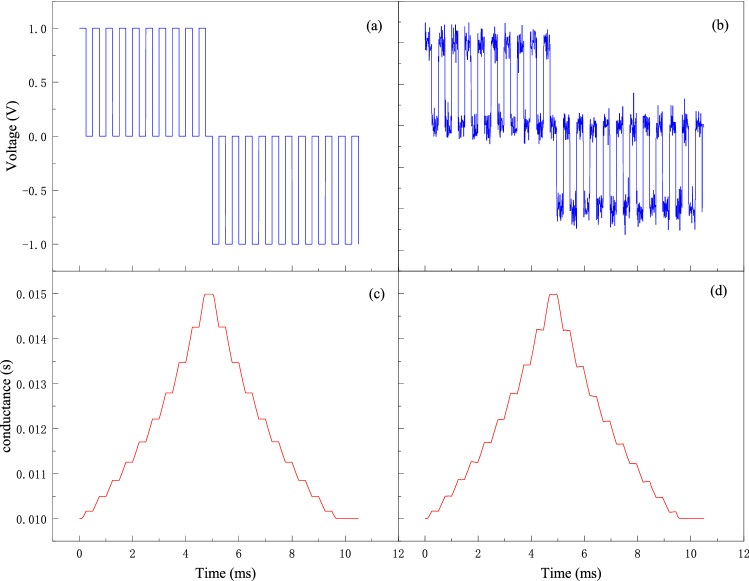
Conductance-voltage (G-V) characteristics of a memristor. (**a**, **c**) without white Gaussian noise; (**b**, **d**) with white Gaussian noise.

**Figure 11:**
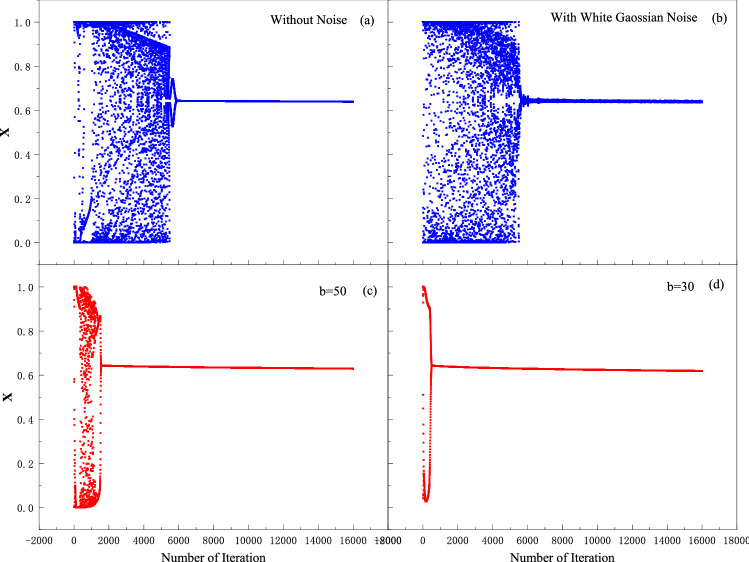
The effect of variability of the memristor on the MTCNN. (**a**) without noise; (**b**) with white Gaussian noise; (**c**) b = 50; (**d**) b = 30.

Here, the impact of the variability of the memristor is discussed based on the proposed memristive neural network. Figure 9 shows the current–voltage (I-V) characteristics of the memristor model^18^. A pinched hysteresis loop is observed when a sinusoidal current is applied to the memristor in Fig. 9a. Figure 9b shows the conductance drift of the memristor from cycle to cycle. The typical conductance-voltage (G-V) characteristics of the memristor model is shown in Fig. 10. Figures 10a and c show when negative voltage pulses are applied to the memristor, the conductance increases gradually, while it decreases when reversed pulses are applied. Figures 10b and d show the white Gaussian noise is added to the device. The white Gaussian noise does not affect the conductance significantly. To further verify the influence of device variability on the chaotic system, we add these changes to the MTCNN. As shown in Figs. 11a and b, the chaotic process of the network after adding Gaussian noise is modified. However, as the chaotic state is still existed, the cryptosystem can still work. The device conductance drift is related to the scaling parameters $b$ of the memristor. The effect of the scaling parameters on the network is shown in Figs. 11c and d. According to Table 2, when the offset of $b$ is within 50%, it only takes effect on the duration of chaos. If the offset is more than 50%, there is no chaos state generated and the network does not work.

The proposed network also shows the good performance under the consideration of the non-idealities of the device and some randomness, which proves it owns strong robustness. This is because unlike the lightweight cryptography, the proposed cryptography does not need absolute stability, and it just needs the general characteristics of memristors. Besides unlike the other improved AES cryptography, it takes the full advantage of the memristor and the chaos to realize "one-time-one-secret" dynamic encryption. This paper presents the prospect of the combination of memristor and encryption, which can significantly improve the safety of the conventional cryptography. However, the proposed system sacrifices some encryption efficiency because of the increase of the computational complexity, and owing to introducing the memristor, it is more difficult for hardware implementation, especially the compatibility of CMOS and memristor.

## Conclusion

In summary, this paper proposes an improved AES cryptosystem based on MTCNN. By using the nonlinear characteristics of a memristor, a memristive neural network is constructed to generate a chaotic sequence with good random characteristics and is applied to improve the key of AES to realize "one-time-one-secret" dynamic encryption. Compared with conventional AES, this algorithm has better performance in image encryption with a more uniform-distribution histogram and a much larger key space. In addition, the proposed AES algorithm has good sensitivity and statistical properties, which can effectively improve the problems of fixed keys and key spaces and can improve the anti-attack ability of the AES algorithm. This paper also fully considers the impact of device variability on the network, and also proposes a circuit level architecture. The hardware implementation of the model in this study with a real memristor and continuous optimization may be carried out in future research.

## Data Availability

The data that support the findings of this study are available from the corresponding author upon reasonable request.
